# CB1 Agonism Alters Addiction-Related Behaviors in Mice Lacking Mu or Delta Opioid Receptors

**DOI:** 10.3389/fpsyt.2018.00630

**Published:** 2018-11-27

**Authors:** Laurie-Anne Roeckel, Dominique Massotte, Mary C. Olmstead, Katia Befort

**Affiliations:** ^1^Laboratoire de Neurosciences Cognitives et Adaptatives (LNCA), Centre de la Recherche Nationale Scientifique, Université de Strasbourg Faculté de Psychologie, Strasbourg, France; ^2^Centre de la Recherche Nationale Scientifique, Université de Strasbourg, Institut des Neurosciences Cellulaires et Intégratives (INCI), Strasbourg, France; ^3^Department of Psychology, Centre for Neuroscience Studies, Queen's University, Kingston, ON, Canada

**Keywords:** ACEA, nociception, withdrawal, depression, anxiety, CB1, opioid

## Abstract

Opioids are powerful analgesics but the clinical utility of these compounds is reduced by aversive outcomes, including the development of affective and substance use disorders. Opioid systems do not function in isolation so understanding how these interact with other neuropharmacological systems could lead to novel therapeutics that minimize withdrawal, tolerance, and emotional dysregulation. The cannabinoid system is an obvious candidate as anatomical, pharmacological, and behavioral studies point to opioid-cannabinoid interactions in the mediation of these processes. The aim of our study is to uncover the role of specific cannabinoid and opioid receptors in addiction-related behaviors, specifically nociception, withdrawal, anxiety, and depression. To do so, we tested the effects of a selective CB1 agonist, arachidonyl-2-chloroethylamide (ACEA), on mouse behavior in tail immersion, naloxone-precipitated withdrawal, light-dark, and splash tests. We examined cannabinoid-opioid interactions in these tests by comparing responses of wildtype (WT) mice to mutant lines lacking either Mu or Delta opioid receptors. ACEA, both acute or repeated injections, had no effect on nociceptive thresholds in WT or Mu knockout (KO) mice suggesting that analgesic properties of CB1 agonists may be restricted to chronic pain conditions. The opioid antagonist, naloxone, induced similar levels of withdrawal in all three genotypes following ACEA treatment, confirming an opioidergic contribution to cannabinoid withdrawal. Anxiety-like responses in the light-dark test were similar across WT and KO lines; neither acute nor repeated ACEA injections modified this behavior. Similarly, administration of the Delta opioid receptor antagonist, naltrindole, alone or in combination with ACEA, did not alter responses of WT mice in the light-dark test. Thus, there may be a dissociation in the effect of pharmacological blockade vs. genetic deletion of Delta opioid receptors on anxiety-like behavior in mice. Finally, our study revealed a biphasic effect of ACEA on depressive-like behavior in the splash test, with a prodepressive state induced by acute exposure, followed by a shift to an anti-depressive state with repeated injections. The initial pro-depressive effect of ACEA was absent in Mu KO mice. In sum, our findings confirm interactions between opioid and cannabinoid systems in withdrawal and reveal reduced depressive-like symptoms with repeated CB1 receptor activation.

## Introduction

Opioid and cannabinoid systems both play a critical role in a number of addiction-related behaviors, such as analgesia, reward, and emotional processing (1, 2). This commonality of function may reflect colocalization of opioid and cannabinoid receptors in brain regions implicated in each process and/or a common mechanism of receptor activation. In terms of the latter, Mu (MOP), Delta (DOP), and Kappa (KOP) opioid receptors, as well as both cannabinoid receptors (CB1 and CB2), are all coupled to inhibitory G proteins. Similarly, anatomical distribution of the three opioid receptors (3, 4) overlaps with CB1 receptor distribution (1) in many areas of the central nervous system (CNS). In contrast, anatomical localization of CB2 receptors in the CNS is not well studied. Indeed, initial reports described CB2 expression in immune cells (5, 6), although more recent work confirmed central expression of these receptors (7–9). Consequently, the anatomical relationship between CB2 receptors and either CB1 or opioid receptors is not known. Importantly, colocalization of opioid and CB1 receptors has been reported in the spinal cord, a critical site for antinociception (10–12), and in higher brain regions associated with emotional processing (13). This pattern of co-expression suggests that cannabinoid-opioid interactions may mediate behavioral responses related to pain relief and addiction (1, 6, 14), a process that may involve the formation of receptor heteromers (15).

Pharmacological and genetic knockout studies confirm interactive effects of opioid and CB1 receptors in antinociception (16) and behaviors related to addiction (17). For example, pharmacological blockade of either opioid or cannabinoid receptors with selective antagonists attenuates behavioral responses induced by an agonist of the other system (14). In addition, genetic inactivation of MOP receptors, producing knockout (KO) mice, decreases physical dependence induced by chronic administration of cannabinoid agonists (18, 19) and reduces the reinforcing properties of these drugs (18). Conversely, inactivation of CB1 receptors inhibits the rewarding properties of a MOP receptor agonist (19–21).

Most studies examining opioid mechanisms in pain relief focus on MOP receptors because of the potent analgesic properties of MOP agonists, such as morphine. Unfortunately, the therapeutic utility of these compounds is often limited as repeated use can lead to both tolerance and addiction (22). One suggestion for increasing clinical efficacy is to combine MOP and cannabinoid agonists, as this leads to increased analgesia (23–25) with fewer side effects (26). Development of these combination drugs depends on a better understanding of opioid-cannabinoid interaction in antinociception and addiction-related behaviors (e.g., tolerance, withdrawal, and emotional processing). To date, the majority of studies examining behavioral responses to cannabinoid receptor activation used Δ9-Tetrahydrocannabinol (THC), the primary phytocannabinoid in the cannabis plant. Because it is a partial agonist at both CB1 and CB2 receptors, THC cannot dissociate the contribution of either cannabinoid receptor to behavioral and affective processes related to pain management. This is a particularly important issue in pain studies, given recent evidence that CB2 receptors are involved in pathological states, such as neuroinflammation and hypersensitivity (27).

In this study, we explored possible interactions between opioid and cannabinoid systems in the mediation of antinociception and addiction-related behaviors. To do so, we examined the effect of the selective CB1 agonist, arachidonyl-2-chloroethylamide (ACEA) (28), on nociception, tolerance, withdrawal, and emotion-related behaviors in MOP or DOP receptor deficient mice. Nociception and tolerance to this effect were assessed in the tail immersion assay; somatic withdrawal symptoms were measured following an injection of the opiate antagonist, naloxone. We relied on naturalistic behaviors to assess anxiety-like (light-dark box) and depressive-like (splash test) responses in mice. Finally, we tested the consequences of both acute and repeated ACEA treatments on opioid-mediated effects in order to assess putative biphasic properties of this agonist.

## Materials and methods

### Animals

One hundred and sixty-eight male and female mice lacking MOP or DOP receptors (MOP KO and DOP KO, respectively) and their wildtype controls (12–24 weeks) were group housed (2–5/cage) under standard light, temperature, and humidity conditions (12 h light-dark cycle, 22 ± 2°C, 55 ± 10% humidity) with *ad libitum access* to food and water. Mice were generated by homologous recombination (29, 30). The genetic background of all mice was 50% C57/BL6J:50% 129svPas.

Research was conducted in accordance with the European Communities Council Directive of 22 September 2010 (directive 2010/63/UE), under the guidelines of the Committee for Research and Ethical issues of the International Association for the Study of Pain (31). Experiments were approved by the local ethics committee (Comité Régional d'Ethique en Matière d'Expérimentation Animale de Strasbourg CREMEAS), and findings are reported following the ARRIVE Guidelines for experiments involving animals.

### Drugs

ACEA (Tocris, Bio-techne, Lille, France) was dissolved in 0.9% saline solution (supplied pre-dissolved in ethanol at 5 mg/ml) to obtain doses of 0.15, 3, and 5 mg/kg. Naloxone (NLX) and Naltrindole (NTI) (Sigma-Aldrich St-Quentin Fallavier, France) were dissolved in saline solution to obtain final doses of 1 and 2.5 mg/kg, respectively. Vehicle (saline or 6% ethanol) injections were used as controls. All drug and vehicle injections were administered ip using 100 μl solution per 10 g bodyweight.

### Behavioral procedures

In each group, approximately equal numbers of male and female mice were used. A total of 22 DOP KO, 49 MOP KO, and 97 WT mice were used. Mice were habituated to the facility and handled for one week before starting the experiments. Behavioral tests were conducted during the light phase and performed blind to genotype and treatment. ACEA or vehicle was administered 45 min before tail immersion or light-dark tests, 35 min before the splash test, and 90 min before precipitated withdrawal.

#### Tail immersion

Thermal nociceptive thresholds were assessed in the tail immersion test by gently restraining mice and immersing ~2/3 of the tail in a water bath at 47°C. The latency to withdraw the tail was recorded before and after ACEA injections. Acute responses to increasing doses of ACEA were tested in WT and MOP KO mice. The effects of repeated ACEA injections (3 mg/kg) on antinociception were tested in a separate group of WT and MOP KO mice by measuring tail immersion responses on day 1 and day 5 of treatment. Hypersensitivity to repeated treatment was assessed 23 h after the last injection, as described previously (32).

#### Naloxone-precipitated withdrawal from ACEA

To evaluate the role of the opioid system in cannabinoid withdrawal, WT, MOP KO, and DOP KO mice received ACEA injections (3 mg/kg once per day for 5 days) followed by a naloxone injection (1 mg/kg), administered 90 min after the last ACEA injection. Withdrawal behaviors were summed over a 20-min observation period and three separate scores were computed for each animal. First, global withdrawal scores were calculated by summing the following values: jumping x 0.8, wet dogs shakes x 1, paw tremors x 0.35, ptosis x 1.5, teeth chattering x 1.5, body tremors x 1.5, and piloerection x 1.5. Second, a subcategory of somatic signs was calculated by combining the total number of jumps, paw tremors, and wet dog shakes. Third, signs of discomfort reflected the sum of stretching, genital licks, and body tremors (33).

#### Light-dark test

The light-dark test, assessing anxiety-like behavior in rodents, employed an apparatus composed of two compartments (20 × 20 × 25 cm), connected by a tunnel (6 × 16.5 × 20 cm) (34). One compartment was brightly illuminated (>400 lx); the other was dark (7 lx). Mice were placed in the dark compartment and allowed to freely explore the apparatus for 5 min while the time spent in each compartment and the tunnel was recorded. Mice have a natural tendency to avoid lit environments: decreased time spent in the dark compartment is a measure of reduced anxiety (35). WT, MOP KO, and DOP KO mice were tested prior to drug administration (baseline; BL) and then following ACEA injections (3 mg/kg) on days 1 and 3. The role of DOP receptors in ACEA-induced changes in anxiety-like behavior was assessed in WT mice by administering NTI prior to ACEA injections; control groups received saline plus ACEA or NTI plus vehicle.

#### Splash test

The splash test (36, 37) consists of vaporizing a 20% sucrose solution on the back fur of mice; mice initiate grooming in response to the solution viscosity. The number of grooming responses (head or body grooming, shakes, and scratches) and the time spent grooming were recorded over 5 min. Repeated stress decreases grooming responses, which is reversed by antidepressant treatment (38), providing an assay for changes in depressive-like behavior in rodents. WT, MOP KO, and DOP KO mice were assessed in the splash test following days 1 and 3 of ACEA treatment (3 mg/kg). As with the light-dark test, WT mice were tested following injections NTI plus ACEA, saline plus ACEA, or NTI plus vehicle.

### Data analyses

Statistical tests were performed using Graphpad Prism® statistical software (Version 6.0; La Jolla, CA, USA). Data from the tail immersion, light-dark, and splash tests were analyzed using a two-way analysis of variance (ANOVA) with genotype as a within subject's factor and dose or day of injection as repeated factors. A 2-way ANOVA (genotype by drug) was used to assess each category of withdrawal score. Subsequent comparisons were conducted using Bonferroni *post hoc* tests. Statistical significance was set at *p* < 0.05.

## Results

Body weight was monitored across the entire experiment with daily weights recorded on all drug treatment days. Using body weight prior to the first injection as a baseline, ANOVA revealed no effect of drug (ACEA, NTI, or NTI + ACEA) on body weights at d5 in either WT (ACEA, 97.5% of BL; NTI, 95.1% of BL, NTI+ACEA, 101.8% of BL), MOP KO (Vehicle, 102.7% of BL; ACEA, 100.7% of BL), or DOP KO (Vehicle, 100.6% of BL; ACEA, 97.9 % of BL) mice.

### Thermal nociception

**Figure 1:**
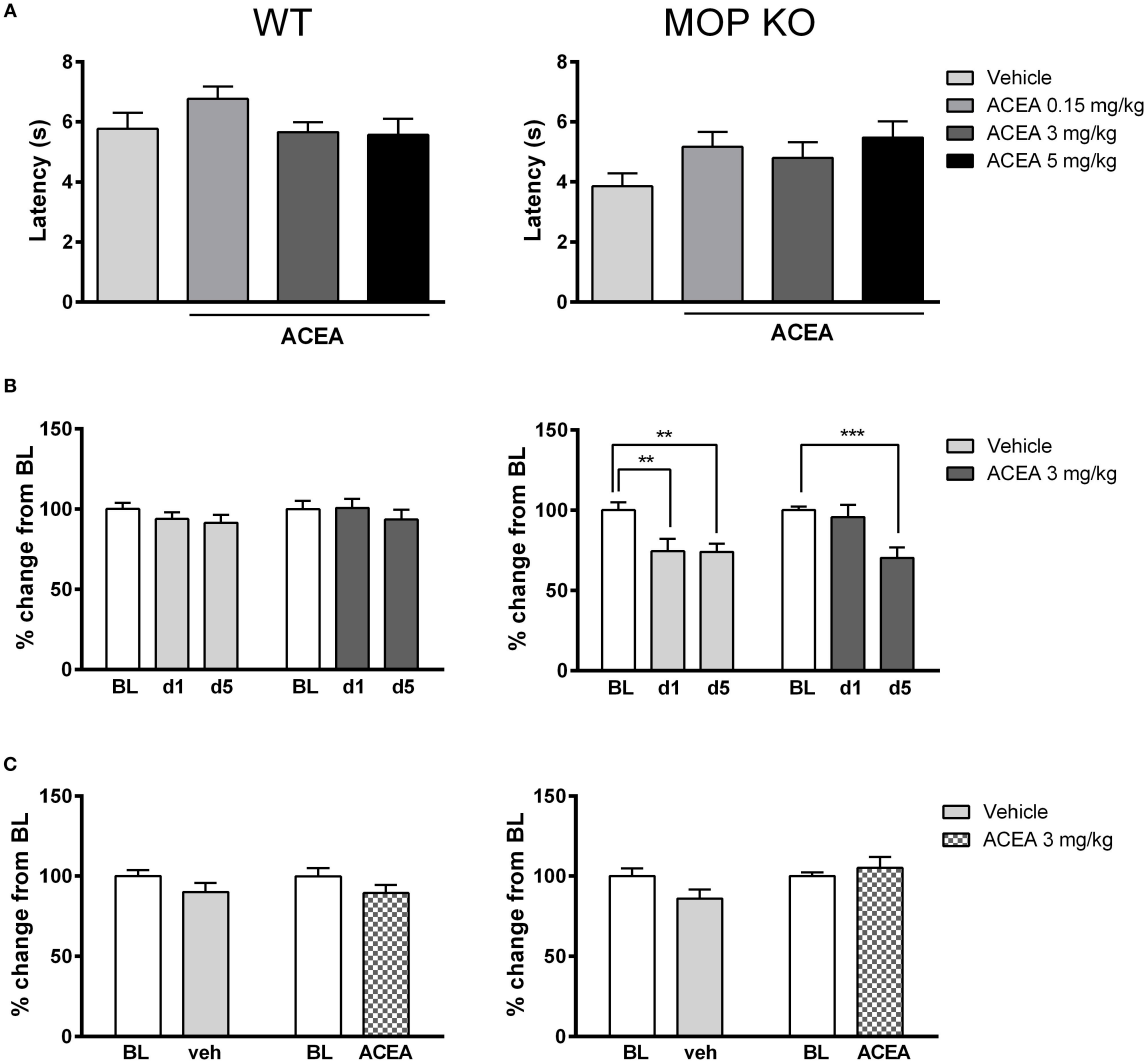
Effects of ACEA on thermal nociceptive thresholds in wildtype (WT) (left) and MOP receptor knockout (KO) (right) mice following acute **(A)** or chronic **(B)** injections and during a post-treatment drug-free test **(C)**. **(A)** Bars represent mean (+SEM) latency (s) to withdraw the tail from a heated bath 45 min after ACEA (0.15, 3, or 5 mg/kg) or vehicle injections. **(B)** Tail withdrawal latencies are expressed as mean (+SEM) % change from baseline (BL) following vehicle or ACEA (3 mg/kg) administration on days 1 and 5 (d1, d5) of a 5-day dosing regime. BL was established one day prior to the first injection. **(C)** Tail withdrawal latencies, shown as mean (+SEM) % change from BL, were assessed 23 h following the final ACEA or vehicle injection. ACEA = arachidonyl-2-chloroethylamide ^**^P < 0.01; ^***^P < 0.001.

As shown in Figure 1A, acute injections of ACEA had no effect on thermal nociception in WT or MOP KO mice across a range of doses [WT: *F*_(3, 32)_ = 1.43, *P* = 0.24; MOP KO: *F*_(3, 31)_ = 1.86, *P* = 0.15]. Although MOP receptors preferentially mediate nociceptive response in mice (30), we did not observe any significant modification of nociceptive thresholds in this genotype, compared to WT mice (*P* > 0.05). We also investigated the effects of repeated subanalgesic doses of ACEA (Figure 1B), revealing no drug-induced alteration in thermal nociceptive thresholds in WT mice [*F*_(1, 45)_ = 0.04, *P* = 0.15] and no effect of repeated injections [*F*_(2, 90)_ = 1.30, *P* = 0.27]. MOR KO mice developed hypersensitivity, with decreased thermal nociceptive thresholds after the 1st vehicle and 5th ACEA injections [Drug X Time Interaction: *F*_(2, 44)_ = 3.40, *P* < 0.05]. Finally, there was no effect of chronic ACEA treatment on thermal nociceptive thresholds in either WT or MOP KO mice, measured 23 h after the last injection (Figure 1C) [WT: *F*_(1, 45)_ = 0.40, *P* = 0.15; MOP KO: *F*_(1, 22)_ = 2.68, *P* = 0.11].

### Naloxone-precipitated withdrawal from ACEA

**Figure 2:**
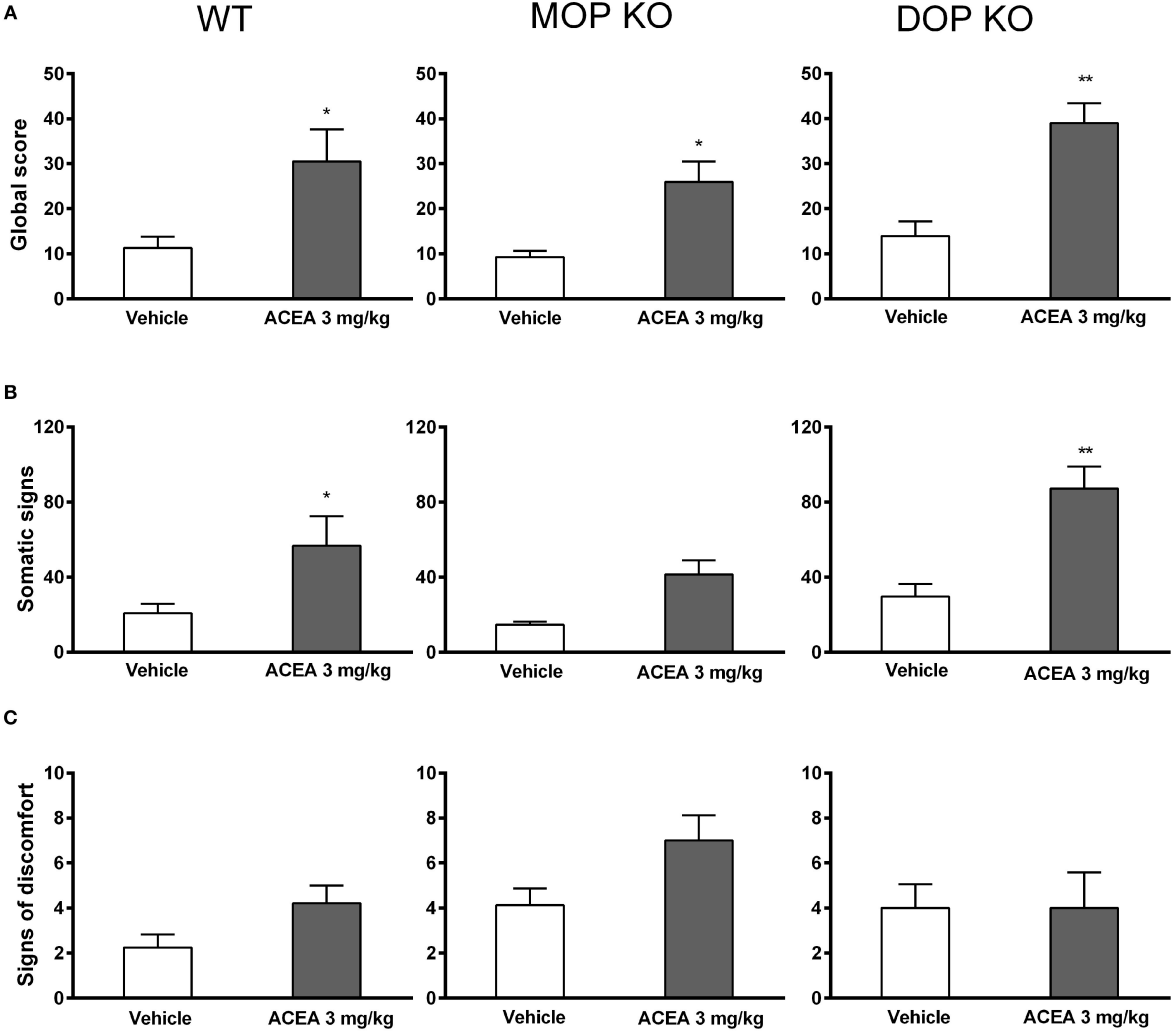
Effects of ACEA treatment on naloxone-precipitated withdrawal in wildtype (WT), MOP knockout (KO), and DOP KO mice. Bars represent mean (+SEM) global withdrawal scores **(A)**, somatic signs **(B)**, and signs of discomfort **(C)** for each genotype over a 20-min observation session. Naloxone (1 mg/kg) was administered 2 h after the last injection of vehicle or ACEA (3 mg/kg per day for 5 days). ACEA = arachidonyl-2-chloroethylamide *P < 0.05; **P < 0.01.

Naloxone induced increased global withdrawal scores in animals chronically treated with ACEA [*F*_(1, 41)_ = 28.85, *P* < 0.0001]. The effect was consistent across WT, MOP KO, and DOP KO mice [*F*_(2, 41)_ = 1.74, *P* = 0.16], with modest changes in all three genotypes treated with vehicle (Figure 2A). A more detailed analysis revealed that ACEA treatment increased both somatic symptoms [*F*_(1, 41)_ = 24.45, *P* < 0.0001] and signs of discomfort [*F*_(141)_ = 3.80, *P* = 0.058], although the latter did not reach statistical significance (Figures 2B,C). Differences in somatic signs in ACEA- and vehicle-treated mice varied across genotype [*F*_(2, 41)_ = 4.50, *P* < 0.05], with MOP KO showing reduced signs of withdrawal compared to WT and DOP KO mice. Signs of discomfort were relatively low in all animals and did not differ across the three lines [*F*_(2, 41)_ = 0.97, *P* = 0.07].

### Light-dark test

**Figure 3:**
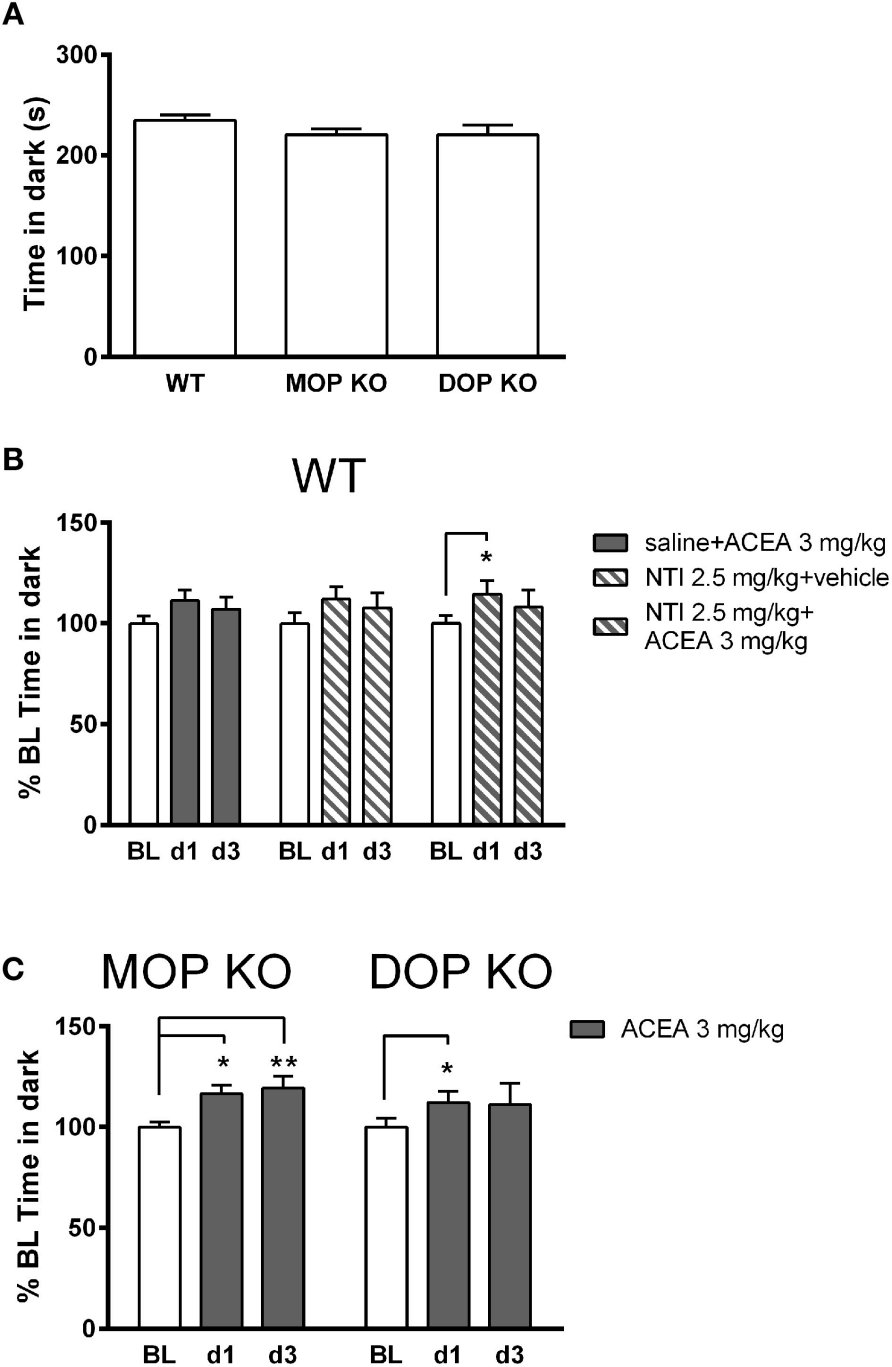
Effects of ACEA treatment on anxiety-like behavior in the light-dark test for wildtype (WT), MOP knockout (KO), and DOP KO mice. **(A)** Bars represent mean (+SEM) time (s) spent in the dark compartment for each genotype over a 5-min drug-free test, which constituted baseline (BL) values. **(B)** Time in the dark compartment is shown as mean (+SEM) % change from BL, assessed on days 1 and 3 (d1, d3) in WT mice, following injections of ACEA plus saline, ACEA combined with naltrindole (NTI), or NTI alone. **(C)** Time in the dark compartment, expressed as mean (+SEM) % change from BL, was assessed in the two KO lines on days 1 and 3 following daily injections of vehicle or ACEA (3 mg/kg). ACEA = arachidonyl-2-chloroethylamide. *P < 0.05; **P < 0.01.

Figure 3A shows that there were no significant differences in baseline levels of anxiety, measured in the light-dark test, across WT, MOP KO, and DOP KO mice [*F*_(2, 37)_ = 1.436, *P* = 0.25]. Repeated drug injections (ACEA with and without NTI) decreased anxiety-like effects in WT mice (Figure 3B) [*F*_(2, 42)_ = 7.93, *P* < 0.01], although there was no overall main effect of drug [*F*_(2, 21)_ = 0.02, *P* = 0.97]. *Post-hoc* tests revealed a significant difference in % time spent in the dark on BL compared to d1 for mice treated with NTI plus ACEA (Bonferroni *post-hoc P* < 0.05). Similarly, repeated injections of ACEA increased % time in the dark in both KO lines [*F*_(2, 28)_ = 8.57, *P* < 0.01] (Figure 3C), due to significant changes from BL to both d1 and d3 in MOP KO mice (Bonferroni *post-hoc P* < 0.05 and *P* < 0.01).

### Splash test

**Figure 4:**
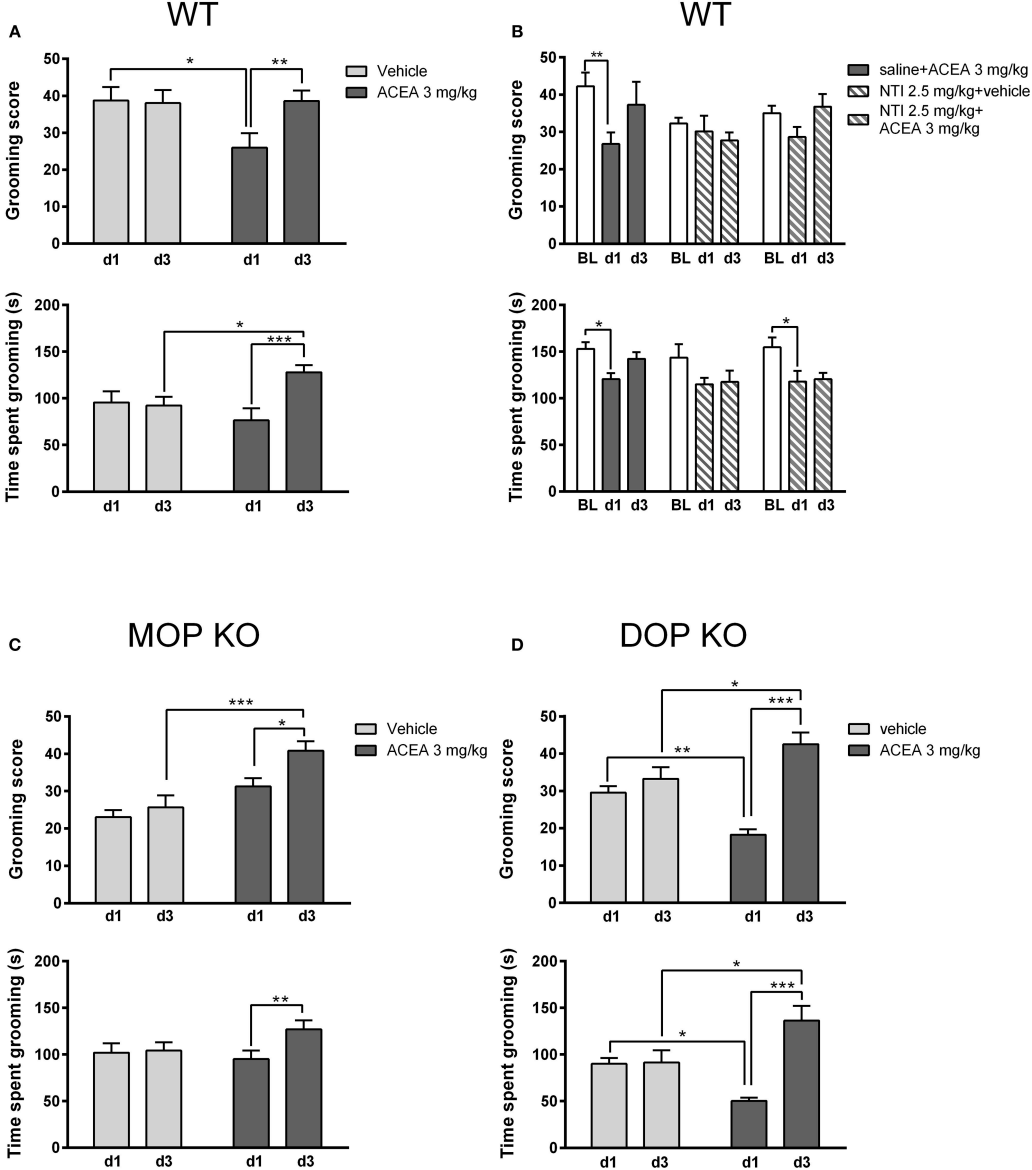
Effects of ACEA treatment on depressive-like behaviors in the splash test for wildtype (WT) **(A)**, MOP knockout (KO) **(C)**, and DOP KO **(D)** mice. Bars represent mean (+SEM) grooming score (left) and time (s) spent grooming (right) in response to vaporization of a 20% sucrose solution on the back fur. Responses were assessed in each genotype over a 5-min period, 45 min after the first and third daily (d1, d3) injections of vehicle or ACEA (3 mg/kg). Grooming responses were assessed in a separate group of WT mice on days 1 and 3 (d1, d3) following injections of ACEA plus saline, ACEA combined with naltrindole (NTI), or NTI alone **(B)**. ACEA = arachidonyl-2-chloroethylamide *P < 0.05; **P < 0.01; ***P < 0.001.

As shown in Figure 4, grooming scores of WT controls (Figure 4A) were higher than both MOP (Figure 4C) and DOP (Figure 4D) KO mice following a vehicle injection, although time spent grooming was consistent across genotypes. Statistical analysis revealed that the effect of ACEA on grooming scores in WT and DOP KO mice was modified with repeated testing [WT: *F*_(1, 23)_ = 7.15, *P* < 0.05; DOP KO: *F*_(1, 12)_ = 15.50, *P* < 0.01]. Analysis of time spent grooming revealed a similar drug x time interaction in these groups [WT: *F*_(1, 23)_ = 15.25, *P* < 0.0001; DOP KO: *F*_(1, 12)_ = 16.23, *P* < 0.01]. These effects were due to decreased grooming on day 1 in ACEA- vs. vehicle-treated mice and increased drug-induced grooming on day 3 compared to day 1 (*post-hoc P*s < 0.05). ACEA increased grooming score [*F*_(1, 29)_ = 17.04, *P* < 0.001], but not time spent grooming [*F*_(1, 29)_ = 0.53, *P* = 0.46], in MOP KO mice. As with WT and DOP KO mice, both measures increased with repeated injections in this group [grooming score: *F*_(1, 29)_ = 7.30, *P* < 0.05; time spent grooming: *F*_(1, 29)_ = 5.38, *P* < 0.05]. In sum, the first ACEA injection decreased grooming in WT and DOP KO mice, while having no effect in MOP KO mice. Interestingly, this prodepressive drug effect was altered following 3 days of injections in WT mice: grooming score returned to basal levels and time spent grooming was increased beyond these levels. The pattern was similar in DOP KO mice, showing increases in both measures following the third ACEA injection. In MOP KO mice, grooming score and time spent grooming were not significantly altered in MOP KO mice on day 1; grooming score increased following the 3rd ACEA injection.

We verified this biphasic effect of ACEA on depressive-like behavior in a separate group of WT mice (Figure 4B), replicating decreased grooming at day 1 with a return to baseline levels by day 3 [grooming score: *F*_(2, 42)_ = 4.41, *P* < 0.05; time spent grooming: *F*_(2, 42)_ = 9.95, *P* < 0.001]. Pretreatment with NTI had no significant effect on either measure [grooming score: *F*_(2, 21)_ = 1.55, *P* = 0.20; time spent grooming: *F*_(2, 21)_ = 1.23, *P* = 0.31].

## Discussion

The goal of this study was to clarify the role of CB1 receptors in addiction-related behaviors and to assess potential interactions with opioid mechanisms, specifically MOP and DOP receptors. Extensive research over the last decades confirms an important contribution of cannabinoid mechanisms to processes such as antinociception, drug dependence, and emotional responses (1, 39, 40), but many of these studies employed THC or other nonselective compounds. Studies using receptor KO mice confirm that both CB1 and CB2 receptors play a role in these processes (1, 41, 42), pointing to the need to examine behavioral effects of pharmacological tools that specifically target each receptor. To this end, we used the selective CB1 agonist, ACEA, as this compound has a Ki of 1.4 nM for CB1, vs. a Ki < 2000 nM for CB2, or 1,400 times greater for CB2 compared to CB1 (43). Confirmation of compound selectivity is provided by abolishment of neurotoxicological effects of ACEA in CB1 KO animals (44), although functional selectivity of G protein signaling may be lost with high doses of ACEA (45).

Use of this highly specific compound revealed a distinct pattern of behavioral effects, some of which differed from results using other CB1 agonists. First, we show that ACEA did not alter bodyweight, despite evidence that other CB1 agonists have orexigenic properties in human and rodents, and that CB1 antagonists may facilitate weight loss (46, 47). We also observed no effect of ACEA on antinociceptive thresholds across a range of doses, contradicting previous evidence that CB agonists such as THC, CP55,940, anandamide, or WIN are analgesic (48, 49). We confirmed a lack of ACEA-induced antinociception in a separate group of mice treated with a single dose and revealed no changes in this measure with repeated injections. It is possible that ACEA doses in our study were too low to be effective as higher doses of THC are required to elicit analgesic responses in the tail immersion, compared to the hot plate, test (20). It is also possible that extending the ACEA dosing regime may have revealed behavioral effects that were not apparent following the protocol used in this study. At the same time, high doses of CB agonists, such as THC, may induce hypolocomotion and catalepsy (50), which could interfere with the behavioral expression of pain and confound measures of antinociception. In addition, THC produces aversive effects at higher doses, such as anxiety and weight loss, which may explain contracictory effects of cannabinoid agonists at high and low doses (51–53). Repeated injections of cannabinoid agonists, such as ACEA, could lead to poor health outcomes minimizing the ethological validity of our findings. Importantly, doses of ACEA comparable to those in our study reduce mechanical allodynia in mouse models of osteoarthritic (54) and neuropathic (55) pain. A more plausible explanation for our negative findings, therefore, is that CB1 receptors are not involved in antinociceptive responses in pain naïve states, fitting evidence that ACEA-induced analgesia in a neuropathic model does not extend to the paw contralateral to the injury (54). This dissociation in the effectiveness of ACEA treatment may reflect altered endocannabinoid signaling, including changes in CB1 receptor function, that is associated with persistent pain states (56).

Our study also provides the first evidence of naloxone-precipitated withdrawal following chronic ACEA treatment. The effect was consistent across all three genotypes but was substantially lower than symptoms observed following treatment with a classic MOP receptor agonist, morphine (33). This general reduction in withdrawal signs may have obscured any genotypic differences in our study, such as decreased withdrawal in MOP or DOP KO mice. If these do exist, they could be revealed by a more intense treatment regime (i.e., higher dose and/or increased number of injections). Regardless, our finding that naloxone elicited withdrawal symptoms in ACEA-treated mice adds to evidence of opioid-cannabinoid interactions in this behavior. For example, cannabinoid antagonists, such as SR141716A, elicit withdrawal symptoms following chronic morphine injections (17, 19, 57) and opioid antagonists induce withdrawal in rodents previously treated with cannabinoid agonists (17). Studies using genetically modified mice confirm opioid-cannabinoid cross-talk in drug withdrawal: naloxone-precipitated withdrawal is decreased in CB1 receptor knockout mice following morphine treatment (19–21) and MOP KO mice exhibit reductions in SR141716A-induced withdrawal following chronic THC treatment (19). The latter effect is dose dependent and alleviated by morphine injections in wildtype mice, providing further support for cannabinoid-opioid interactions in drug withdrawal.

Given the reciprocal relationship between opioid and cannabinoid systems in drug withdrawal, our observation that ACEA treatment induced withdrawal symptoms in both MOP and DOP KO mice seems counterintuitive. These findings could suggest that the KOP receptor has a critical role in cannabinoid withdrawal. This fits evidence that DOP receptors contribute to negative affective states induced by THC (18, 20, 58), but have no role in the anxiolytic properties of the drug (59). In addition, naloxone is a nonselective antagonist so if a single receptor subtype is deleted (e.g., MOP or DOP), withdrawal symptoms could be elicited through an action at the remaining, intact receptors. This would explain why naloxone-precipitated withdrawal following cannabinoid treatment is not modulated in mice lacking either MOP, DOP, or KOP receptors (18).

A novel finding in our study is that initial administration of a CB1 agonist produces a depressive-like state, which recovers with further drug exposure. This could explain why the rewarding effects of cannabinoid agonists, including THC, are only revealed in self-administration or place conditioning paradigms when animals receive priming injections prior to training (18, 51, 53, 60, 61). Interestingly, the pro-depressive effect of acute ACEA injections (i.e., day 1) was absent in MOP KO mice, mimicking decreased depressive-like symptoms of this genotype in other behavioral tests (29). Despite this initial blunting, repeated ACEA injections reduced depressive-like symptoms in MOP KO animals, matching behavioral effects observed in DOP KO mice. WT mice showed a different profile: repeated ACEA injections restored grooming scores to basal levels but increased time spent grooming above control levels. MOP receptors, therefore, appear to mediate the initial pro-depressive effects of ACEA without affecting the subsequent anti-depressive effects of repeated exposure.

In contrast to depressive-like behaviors, we observed no effect of either acute or chronic ACEA injections on anxiety-like responses in the light/dark box. This appears to contradict previous findings in the elevated plus maze (54), although decreased time spent in open arms was only observed with higher doses of the drug. At least for another CB agonist (THC), anxiolytic properties at low doses (59, 61) are replaced by anxiogenic effects at higher ones (51, 61). As noted previously, hypolocomotion and catalepsy induced by higher dose of THC (50) could modify responses in a number of behavioral tasks including the light-dark test, open field, and elevated plus maze. At the very least, given that we used identical dosing procedures in splash and light/dark tests, our findings provide evidence for a dissociation in the effects of CB1 activation on anxiety- and depressive-like responses in mice.

Given the key role of DOP receptors in anxiety and depression (29), we went on to test whether pharmacological blockade of these receptors in WT mice would alter responses in splash or light/dark tests. The DOP receptor antagonist, NTI, was ineffective on its own, but decreased time spent grooming in the splash test when combined with ACEA. This reduction was not matched by a reduction in grooming score, suggesting that the drug combination may have disrupted a general pattern of naturalistic behavior (i.e., grooming). Overall, our results are consistent with previous studies: although NTI disrupts pro-depressive or anxiogenic effects of DOP agonists (62–64), it is ineffective when administered alone (65, 66). Our findings that pharmacological blockade of DOP receptors has no effect on anxiety- or depressive-like behaviors conflicts with studies using genetically modified mice (67), suggesting that developmental adaptations impact the function of these receptors in emotional expression.

In sum, our results help to clarify how opioid and cannabinoid systems interact in behavioral processes associated with addiction. The use of a selective CB1 agonist revealed no involvement of this receptor in either antinociception or anxiety-like behaviors in mice. In contrast, repeated activation of CB1 receptors induced opioid-dependent withdrawal symptoms and produced a biphasic effect on depressive-like symptoms. In the long-term, this information could facilitate the development of new pain medications that reduce the incidence of affective and substance use disorders that currently characterize long-term opioid use.

## Author contributions

L-AR and KB designed and performed the experiments and wrote the manuscript. L-AR collected data and analyzed the results under the guidance of KB. DM provided the animals and consulted on the design of the experiments. MO contributed to the discussion of the data and writing of the manuscript.

### Conflict of interest statement

The authors declare that the research was conducted in the absence of any commercial or financial relationships that could be construed as a potential conflict of interest.

## Abbreviations

ACEA: arachidonyl-2-chloroethylamide
CNS: central nervous system
DOP: delta opioid
KO: knockout
KOP: kappa opioid
MOP: mu opioid
NLX: naloxone
NTI: naltrindole
THC: Δ9-Tetrahydrocannabinol
WT: wildtype.
